# A Risk Model of Eight Immune-Related Genes Predicting Prognostic Response to Immune Therapies for Gastric Cancer

**DOI:** 10.3390/genes13050720

**Published:** 2022-04-20

**Authors:** Miao Yu, Yi Zhang, Rongchen Mao, Chao Zhu, Ruixue Zhao, Lai Jin

**Affiliations:** 1 The First School of Clinical Medicine, Nanjing Medical University, Nanjing 210029, China; annebee@163.com; 2 College of Computer Science and Technology, Zhejiang University, Hangzhou 310027, China; z1@zju.edu.cn; 3 School of Basic Medical Sciences, Nanjing Medical University, Nanjing 210029, China; mrc6276@126.com (R.M.); czhu@njmu.edu.cn (C.Z.); 4 School of Biomedical Engineering and Informatics, Nanjing Medical University, Nanjing 210029, China; zhaoruixue@njmu.edu.cn

**Keywords:** immune-related genes, prognostic model, WGCNA, gastric cancer

## Abstract

Immune checkpoint inhibitor (ICI) treatment is considered as an innovative approach for cancers. Since not every patient responded well to ICI therapy, it is imperative to screen out novel signatures to predict prognosis. Based on 407 gastric cancer (GC) samples retrieved from The Cancer Genome Atlas (TCGA), 36 immune-related hub genes were identified by weighted gene co-expression network analysis (WGCNA), and eight of them (*RNASE2*, *CGB5*, *INHBE*, *DUSP1*, *APOA1*, *CD36*, *PTGER3*, *CTLA4*) were used to formulate the Cox regression model. The obtained risk score was proven to be significantly correlated with overall survival (OS), consistent with the consequence of the Gene Expression Omnibus (GEO) cohort (*n* = 433). Then, the relationship between the risk score and clinical, molecular and immune characteristics was further investigated. Results showed that the low-risk subgroup exhibited higher mutation rate, more M1 macrophages, CD8^+^ and CD4^+^ T cells infiltrating, more active MHC-I, and bias to “IFN-γ Dominant” immune type, which is consistent with our current understanding of tumor prognostic risk. Furthermore, it is suggested that our model can accurately predict 1-, 2-, and 3-year OS of GC patients, and that it was superior to other canonical models, such as TIDE and TIS. Thus, these eight genes are probably considered as potential signatures to predict prognosis and to distinguish patient benefit from ICI, serving as a guiding individualized immunotherapy.

## 1. Introduction

Gastric cancer (GC) is the second most lethal cancer worldwide, and the incidence rate is higher in East Asia [1]. Although complete resection is the only chance of cure, recurrence is common. To avoid the possibility of relapse, perioperative chemotherapy is essential for GC. Currently, immune checkpoint inhibitor (ICI) treatment such as anti-cytotoxic T-lymphocyte antigen 4 (*CTLA4*) mAb and anti-programmed death-1 (PD-1) mAbs is considered as an innovative treatment strategy for advanced GC [2]. The ICI treatment for GC has shown significant benefits in survival [3]. However, ICI treatment seems to be more effective for the subgroups with high mutation burden, Epstein-Barr virus (EBV) positive, or microsatellite instability high [4]. Multiple factors including the tumor immune microenvironment (TME) influence ICI effectiveness, and few accurate biomarkers can predict the response to ICI [5]. Identification of potential prognostic markers and the development of ICI guidelines can allow for individualization of the immunotherapy for patients with GC. Some researchers suggested that a deeper analysis of TME complexity was helpful for revealing advanced biomarkers that identified patient populations responsive to ICI therapy [6]. Unfortunately, we still know little about the TME of GC, and effective prognostic signatures are urgently needed.

In this study, we sought to develop a series of biomarkers for the immune profile of GC, which may contribute to immune therapy choice. We originally focused on the differentially expressed genes between normal (*n* = 32) and tumor (*n* = 375) samples from TCGA and screened out immune-related hub genes through WGCNA. Next, eight genes were identified to build a prognostic model, which was verified by the GC samples from GEO (*n* = 433). Then, to further investigate the model, we analyzed the molecular and immune characteristics of the high-risk (HR) and low-risk (LR) subgroups and discussed their immune efficacy. Finally, we calculated the accuracy of our model in predicting gastric cancer prognosis and compared the advantages and disadvantages of our model and several existing models, such as the immune dysfunction and exclusion (TIDE) model and the tumor inflammation signature (TIS) model. The results revealed that our model provided promising prognostic biomarkers for immune subtypes that guided patients receiving immune therapy.

## 2. Methods

### 2.1. Data Collection and Processing

The detailed information of GC samples was downloaded from The Cancer Genome Atlas—Stomach Adenocarcinoma (TCGA-STAD), including 407 RNA sequencing (RNA-seq) data (375 cancer samples and 32 para-cancer samples) and 443 clinical information (https://portal.gdc.cancer.gov, accessed on 24 August 2021). Then, we used the limma package (limma powers differential expression analyses for RNA-sequencing and microarray studies. Nucleic Acids Research 43(7), e47) of R to merge transcriptome profiling and clinical data to conduct follow-up analysis. The data of nucleotide mutation in these specimens were also downloaded from TCGA-STAD, Workflow Type: VarScan2 Annotation.

Another 433 GC samples used to validate our model (GSE84437) were downloaded from another database, Gene Expression Omnibus (GEO: https://www.ncbi.nlm.nih.gov/geo/, accessed on 24 August 2021), comprising RNA-seq data and their survival information and data processing described above.

The immune-related genes used in our study were downloaded from the Immunology Database and Analysis Portal (ImmPort) (https://www.immport.org/shared/home, accessed on 24 August 2021) and InnateDB (https://www.innatedb.com/, accessed on 24 August 2021) databases.

The immune subtypes of the 371 GC samples were provided by USUC Xena (https://xena.ucsc.edu, accessed on 24 August 2021). The specific data of immunotherapy outcome-related scores (TIDE, MSI, exclusion, and dysfunction situation) were obtained from Tumor Immune Dysfunction and Exclusion (TIDE: http://tide.dfci.harvard.edu/, accessed on 24 August 2021).

### 2.2. Identification of Prognostic Immune-Related Genes

To look for genes with differential levels in normal and tumor samples, we used the limma package of R (fdr (false discovery rate) < 0.05, |log2FC| > 1), based on TCGA RNA-seq data (375 tumor and 32 normal). Then, we took the intersection of these genes with immune-related genes obtained from ImmPort and InnateDB for identifying differentially expressed immune-related genes. For these genes obtained above, Gene Ontology (GO) and Kyoto Encyclopedia of Genes and Genomes (KEGG) analyses were performed with the R software “clusterProfiler” package 3.14 (https://bioconductor.org/packages/release/bioc/html/clusterProfiler.html, accessed on 24 August 2021). (q value < 0.05) and the “ggplot2” (ggplot2: Elegant Graphics for Data Analysis. Springer-Verlag New York, 2016) and “GOplot” (GOplot: an R package for visually combining expression data with functional analysis. Bioinformatics (2015): btv300) packages in R were used for visualization of GO and KEGG enrichment analysis results.

To identify hub genes in connection with the clinical state of GC, the weighted gene co-expression network analysis (WGCNA) was performed based on the WGCNA package of R. First, we preprocessed the expression data of differentially expressed immune genes (*n* = 493) and deleted those with little fluctuation in expression. Next, we clustered samples to exclude outliers and prepared the non-outliers for the next step. Then, we obtained the optimal power value from the fitting index and average connectivity with the power scatter plot. Under this power value, we can obtain the optimal scale-free network. Then, the similarity matrix was constructed by calculating the Pearson coefficient between any two genes, which was transformed into an adjacency matrix based on the optimal power value (a soft threshold of β = 3). Next, a topological matrix was also constructed to describe the degree of association between genes with the topological overlap measure (TOM) (1-TOM was used as the distance to cluster the genes). Finally, gene modules were determined by the dynamic cut tree method, and those modules with high similarity were merged (height = 0.25).

Since the genes in the turquoise modules were the most significantly correlated to the clinical trait (normal vs. tumor) (*p* = 4 × 10^−34^), they were used to construct the co-expression network (weight threshold > 0.3). Their gene expression quantity was then extracted from the tumor samples of TCGA (*n* = 375) and combined with the survival data (*n* = 371), using the limma package of R. Thus, 36 prognostic genes (*p* < 0.05) were obtained by univariate COX analysis, and their survival curves were made based on the best cut-off value, obtained by the surv_cutpoint function from survminer (survminer: Drawing Survival Curves using ‘ggplot2’. R package version 0.4.9. https://CRAN.R-project.org/package=survminer, accessed on 24 August 2021) package of R.

### 2.3. Construction and Validation of the Risk-Score Model

Among the 36 immune-related genes, 8 genes significantly affecting survival were identified based on the 371 GC samples from TCGA, to construct a prognostic model by multivariable Cox regression analysis. The risk score of each sample was calculated by adding the product of each genes’ expression and their certain coefficient (obtained from survival package of R, Therneau T (2021). _A Package for Survival Analysis in R_. R package, version 3.2-13. https://CRAN.R-project.org/package=survival, accessed on 24 August 2021). The formula is as follows: $\mathrm{Riskscore}=\sum_{i=1}^{8}\left(Gene_{i}\times Coef_{i}\right)$.

Then, Kaplan–Meier (K–M) survival curves were drawn with log-rank tests using both TCGA and GEO cohorts to evaluate the prognostic power of the model. Furthermore, univariate and multivariable Cox regression analyses were also performed to validate the independent prognostic value of the model.

### 2.4. Comprehensive Analysis of Molecular and Immune Characteristics in HR and LR Subgroups

The gene set enrichment analysis (GSEA) was applied to study the enrichment pathways and functions of differentially expressed genes (using the limma package of R) between HR (*n* = 185) and LR (*n* = 186) subgroups (*p* < 0.05). Two reference gene sets used in our research were “KEGG sets as Gene Symbols” (http://www.gsea-msigdb.org/gsea/msigdb/download_file.jsp?filePath=/msigdb/release/7.5.1/c2.cp.kegg.v7.5.1.symbols.gmt, accessed on 24 August 2021) and “all GO gene sets as Gene Symbols” (http://www.gsea-msigdb.org/gsea/msigdb/download_file.jsp?filePath=/msigdb/release/7.5.1/c5.go.v7.5.1.symbols.gmt, accessed on 24 August 2021), and the clusterProfiler package of R was applied. In the gene mutation analysis, the Maftools package of R was used to calculate and collate mutation data of HR and LR samples, and the genes with the top 20 mutation rates were exported. Then, correlation analysis between risk score and 4 targeting genes (*PD-L1*, *CTLA4*, *TGFB1*, *CXCL12*) and total mutation burden (TMB) was performed.

In the immune characteristic analysis, the gene expression data from TCGA (*n* = 407) were adopted to explore immune cell infiltration. We applied CIBERSORT (Cell type Identification by Estimating Relative Subsets of RNA Transcripts, https://cibersortx.stanford.edu, accessed on 24 August 2021) to calculate the fractions of the 22 immune cell types based on the default signature matrix at 1000 permutations. Then, the boxplot and barplot were drawn based on the limma package of R to see if the proportion of immune cells varied between HR and LR subgroups. Next, based on the best cut-off obtained by surv_cutpoint, we compared the survival differences between high- and low-expression groups of immune cells. Furthermore, to investigate the relationship between GC prognosis and immunity, we scored each sample for immune function. For this purpose, single sample GSEA (ssGSEA) analysis and correction were performed relying on several representative gene sets with the GSVA package (http://www.bioconductor.org/packages/release/bioc/html/GSVA.html, accessed on 24 August 2021) of R. Next, to explore differences in survival, K–M survival curves were applied.

By uploading expressed data files onto the TIDE website, we finally obtained the TIDE relevant scores of each sample (*n* = 407).

### 2.5. Verifying the Model Accuracy and Contrasting It with Traditional Ones

In this section, time-dependent receiver operating characteristic (ROC) curve analysis was applied to calculate the value of area under the curve (AUC) with the timeROC package of R (https://cran.r-project.org/web/packages/timeROC/index.html, accessed on 24 August 2021). Then we obtained the AUC in predicting 1-, 2- and 3-year overall survival (OS) separately. To prove the superiority of our model, a comparison of our prognostic model and other classical models (TIDE model and TIS model) was also conducted.

### 2.6. Statistical Analysis

Continuous variables were compared between the two subgroups by the independent T test, classified data were compared by the chi-square (χ^2^) test, and the TIDE score was compared by the rank sum test. Univariate survival analysis was performed by K–M survival analysis with the log-rank test, while the Cox regression was used to carry out multivariable survival analysis. A two-sided *p* < 0.05 was considered significant and marked for ‘*’, *p* < 0.01 (**), *p* < 0.001 (***), and *p* > 0.05 (ns).

## 3. Results

### 3.1. Outcomes of Prognostic Immune-Related Genes

**Figure 1:**
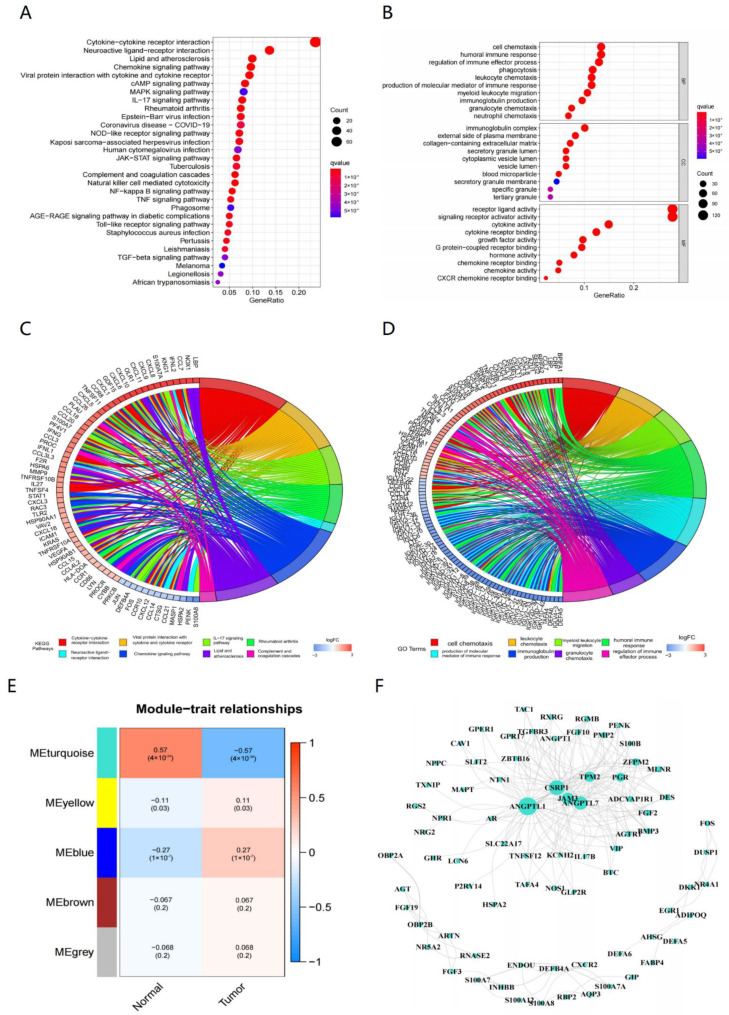
GSEA of immune-related differentially expressed genes (IDEG). (**A**) Top 30 Kyoto Encyclopedia of Genes and Genomes (KEGG) pathways enriched in the IDEG (*p* < 0.05). (**B**) Top 10 biology processes (BP), cellular components (CC) and molecular functions (MF) in Gene Ontology (GO) enrichment analysis enriched in the IDEG (*p* < 0.05). (**C**) Circos of the GO analysis outcomes. (**D**) Circos of the KEGG analysis outcomes. (**E**) Gene modules related to GC obtained by WGCNA. (**F**) The network of the genes in the turquoise module (weight > 0.3).

A list of 8833 genes was identified by differential expression analysis based on 375 tumor samples and 32 normal samples (Supplementary Figure S1A), among which 493 were immune-related differentially expressed genes. Of those immune-related genes, 309 genes were upregulated and 184 were downregulated in the tumor samples compared with normal samples (Supplementary Figure S1B). In GO and KEGG analyses, we found that 69 pathways and 1688 GO terms, including 1561 biology processes (BP), 29 cellular components (CC), 98 molecular functions (MF), were significantly enriched in these 493 differentially expressed immune-related genes. The corresponding bubble plots of the top 30 KEGG pathways and top 10 BP, CC, MF are shown in Figure 1A,B. Circos are also shown in Figure 1C,D.

To obtain the immune-related prognostic genes, we first obtained the sample dendrogram and heatmap based on WGCNA analysis (Supplementary Figure S2A) The changes in the network regression slope and connectivity under different β are shown in Supplementary Figure S2B; the optimal power value was 3 and its *R* = 0.90649810, slope = −1.3467096. Then, the dynamic cut tree algorithm was used to perform preliminary clustering of the topology matrix, and the elementary network modules were identified. Next, we merged the network modules with high similarity (Height = 0.25), with a total of five modules (Supplementary Figure S2C). The final results of module eigengenes clustering are drawn as Supplementary Figure S2D.

**Figure 2:**
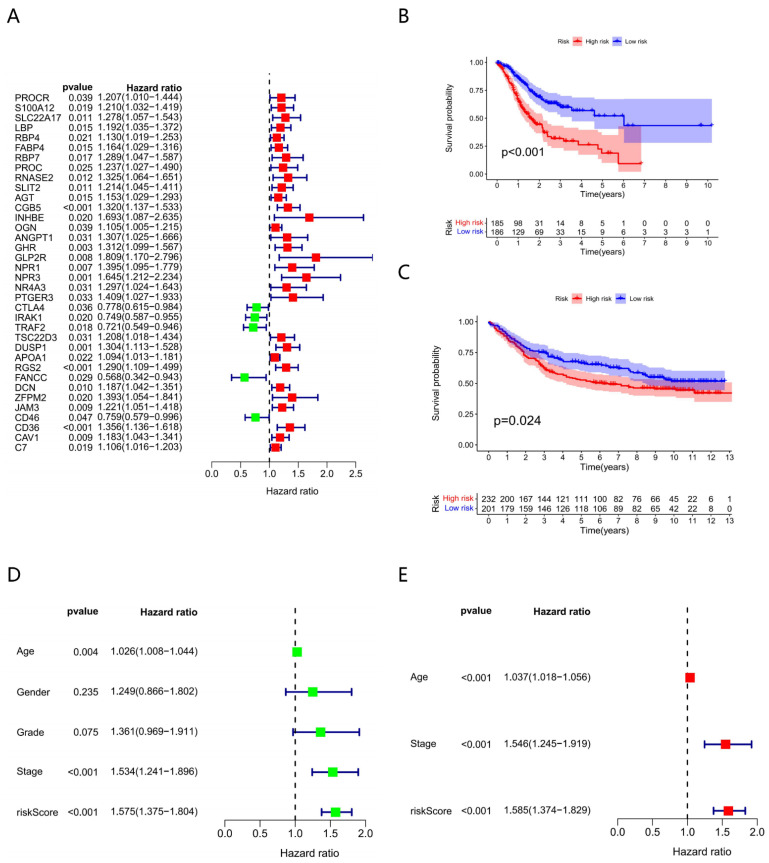
Construction and validation of the risk-score model. (**A**) Univariate Cox analysis of 36 immune-related prognostic genes. (**B**) The Kaplan–Meier (K–M) survival curve of different subgroups in the TCGA cohort. (185 high-risk vs. 186 low-risk). (**C**) The Kaplan–Meier (K–M) survival curve of different subgroups in the GEO cohort (232 high risk vs. 201 low risk). (**D**) Univariate Cox analysis of clinical factors and the risk score (*p* < 0.001). (**E**) Multivariable Cox analysis of clinical factors and the risk score (*p* < 0.001).

Thus, the relationship of module and trait was calculated and is shown in Figure 1E, which indicates that there were three modules closely correlated with GC (*p* < 0.05), and the turquoise one was the most significant; the genes in these modules were selected for further analysis. There were 78 genes and 204 edges for the turquoise module of the networks with a threshold weight > 0.3 (Figure 1F). Thirty-six prognostic genes (*p* < 0.05) were closely correlated with GC patient OS as determined by K–M analysis, among which 31 genes were considered as HR-related genes (HR > 1) and five were as LR (HR < 1) (Figure 2A). Then, according to the best cut-off value of each gene, the GC samples were divided into high- and low-expression subgroups and obtained the corresponding survival curves (Supplementary Figure S4). Furthermore, we measured the mutation rate of each gene in TCGA samples (*n* = 431), with *SLIT2* as the highest (Supplementary Figure S3).

### 3.2. Constructing Model via 8 Biomarkers

Eight genes were identified as prognostic biomarkers to carry out the risk score (Table 1). Among them, *PTGER3* and *CTLA4* were strongly inversely associated with the prognostic risk, and *RNASE2*, *CGB5*, *INHBE*, *DUSP1*, *APOA1*, *CD36* had a positive impact on risk. Their corresponding coefficients from survival R package are demonstrated in table (Table 1).

To further explore the mechanism of risk score influencing prognosis, we investigated the clinical, molecular, and immunogenetic features of different scoring subgroups.

### 3.3. Clinical Characteristics of Different Risk Subgroups

We assigned patients to the HR and LR subgroups using median risk scores, and survival analysis was performed on both subgroups, which showed that patients of the LR subgroup had a better OS than that of the HR subgroup (*p* < 0.001) (Figure 2B). Meanwhile, the GSE84437 GC dataset (*n* = 433) for survival analysis made the model more convincing, and the results were consistent; the prognosis of the LR subgroup was better (*p* < 0.05) (Figure 2C).

However, other clinical features such as age, gender, grade, stage, and TMN were evenly distributed between HR and LR subgroups; the results are presented in the heatmap (Supplementary Figure S5).

In univariate and multivariable Cox regression analyses, we found that age and stage were significantly associated with the prognosis of GC, and risk score was confirmed as an independent prognostic factor (*p* < 0.001) (Figure 2D,E).

### 3.4. Molecular Characteristics of Different Risk Subgroups

**Figure 3:**
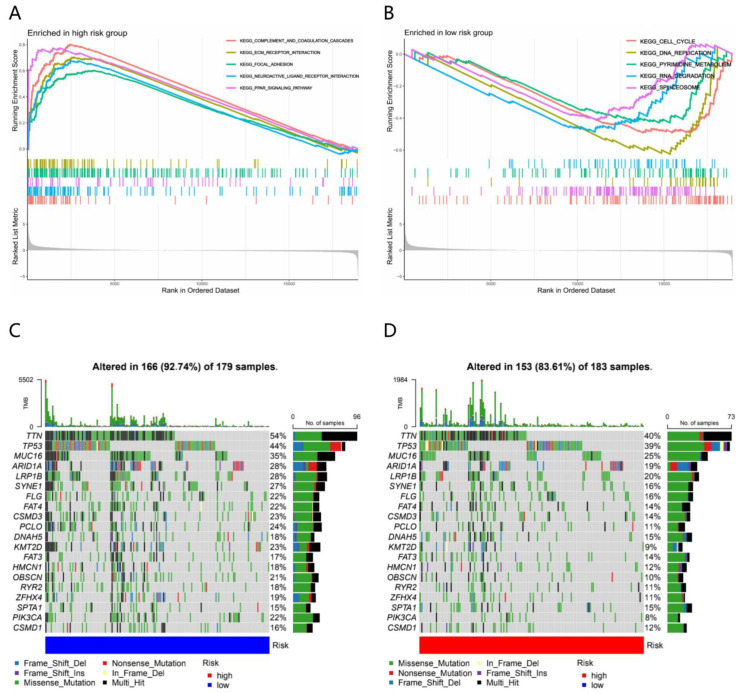

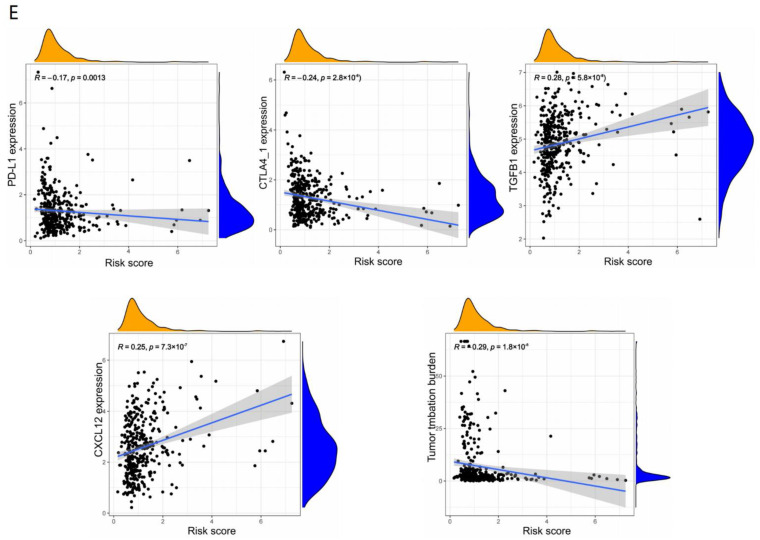
Molecular characteristics of different risk subgroups (**A**) The top 5 enrichment pathways enriched in the high-risk subgroup (*p* < 0.05) obtained by gene set enrichment analysis (GSEA). (**B**) The top 5 enrichment pathways enriched in the low-risk subgroup (*p* < 0.05) obtained by GSEA. (**C**) The oncoplot showing the significantly mutated genes in the low-risk GC samples (top 20). (**D**) The oncoplot showing the significantly mutated genes in the high-risk GC samples (top 20). (**E**) The relationship between risk score and *PD-L1*, *CTLA4*, *TGFB1*, *CXCL12* expression and TMB.

GSEA was performed to determine the gene sets enriched in different risk subgroups. The gene sets of the HR samples were significantly enriched in 41 pathways (*p* < 0.05), especially in complement and coagulation cascades pathway (NES = 2.25) (Figure 3A), while the gene sets of the LR samples were significantly enriched in 14 pathways (*p* < 0.05), especially in DNA replication pathway (NES = −1.56) (Figure 3B).

Gene mutations were then analyzed to gain further biological insight into the TME of different risk subgroups. We found that the mutation rate in the LR subgroup (altered in 166 (92.74%) of 179 samples) was significantly higher than that in the HR subgroup (altered in 153 (83.61%) of 183 samples). Missense mutation was the most common mutation type, followed by nonsense and frame shift deletions. Genes with the highest mutation frequency were TTN (54% in LR samples and 40% in HR samples), TP53 (44% in LR samples and 39% in HR samples), MUC16 (35% in LR samples and 25% in HR samples) and so on (Figure 3C,D).

Next, we explored the relationship between risk score and the expression of those of therapeutic targeting (*PD-L1*, *CTLA4*, *TGFB1*, *CXCL12*) and TMB. Genetic difference analysis indicated that the expression of PD-L1 (*R* = −0.17; *p* < 0.01) and *CTLA4* (*R* = −0.24; *p* < 0.001) ere distinctly higher in the LR subgroup than in the HR subgroup (Figure 3E), while TGFB1 (R = 0.28; *p* < 0.001) and *CXCL12* (*R* = 0.25; *p* < 0.001) were lower in the LR subgroup (Figure 3E). TMB was negatively correlated with risk score (*R* = −0.29; *p* < 0.001) (Figure 3E), which means that higher TMB may represent more sensitivity to treatment.

### 3.5. Immune Characteristics of Different Risk Subgroups

**Figure 4:**
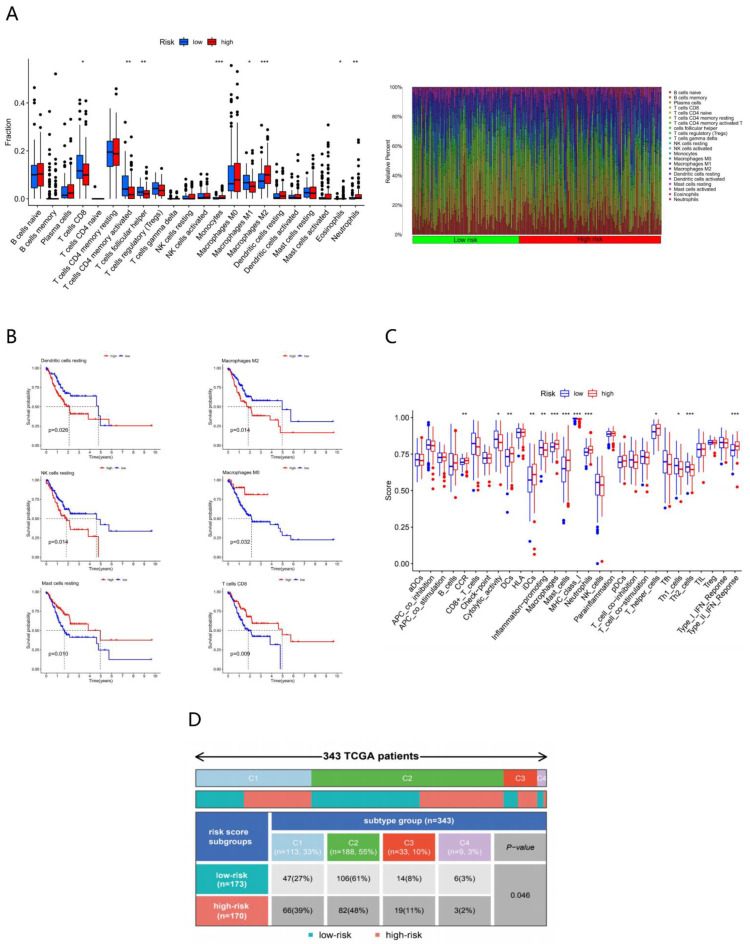

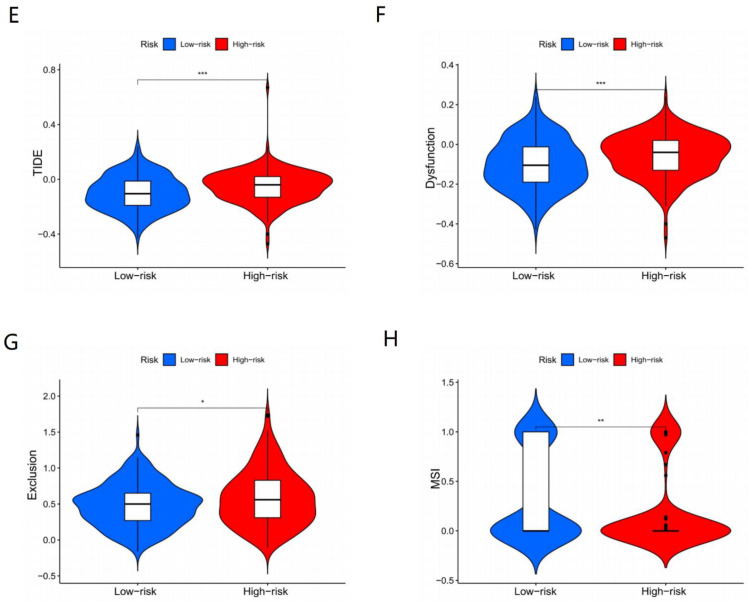
Immune characteristics of different risk subgroups (‘*’ stands for *p* < 0.05, ‘**’ stands for *p* < 0.01, ‘***’ stands for *p* < 0.001). (**A**) The infiltration of 22 subtype immune cells in high- and low-risk subgroups. (**B**) K–M survival analysis of the 6 differential infiltration immune cells. (**C**) The score of 29 immune functions. (**D**) Heatmap and table showing the distribution of immune subtypes between the different risk subgroups (χ^2^ = 0.046). Immunotherapy effects of different subgroups: (**E**) TIDE, (**F**) T-cell exclusion and (**G**) dysfunction, (**H**) MSI score in different risk subgroups.

The infiltration of immune cells in each sample is displayed in Figure 4A. Through differential analysis, we found that the proportion of T cells CD4 memory activated, T cells follicular helper (*p* < 0.01), T cells CD8^+^ and Macrophages M1 (*p* < 0.05) was higher in the LR subgroup, while the content of Monocytes, Macrophages M2 (*p* < 0.001), Neutrophils (*p* < 0.01) and Eosinophils (*p* < 0.05) was more abundant in the HR subgroup. Taking the median expression as the cut-off value, we could find that low expression of dendritic cells resting, Macrophages M2 and NK cells resting was associated with high survival rate, but low expression of T cells CD8, Macrophages M0, and Mast cells resting was associated with low survival rate (*p* < 0.05) (Figure 4B).

Next, to penetrate into the immune patterns of different risk subgroups, we marked 29 immune-related functions of each sample via ssGSEA. Thirteen of them showed differences between the HR and LR subgroups: CCR (*p* < 0.01), DCs (*p* < 0.01), iDCs (*p* < 0.01), Macrophages (*p* < 0,001), Mast cells (*p* < 0.001), Neutrophils (*p* < 0.001), T helper cells (*p* < 0.05), Type II IFN Response (*p* < 0.001) were active in the HR subgroup; however, Cytolytic activity (*p* < 0.05), Inflammation−promoting (*p* < 0.01), MHC class I (*p* < 0.001), Th1 cells (*p* < 0.05), Th2 cells (*p* < 0.001) were relatively silent in the LR subgroup (Figure 4C), which indicated that these immune-related functions were closely linked with the prognosis.

Furthermore, the 343 TCGA samples were classified according to a novel immune landscape of cancer, which identified tumor as six immune subtypes: Wound Healing (C1), IFN-γ Dominant (C2), Inflammatory (C3), Lymphocyte Depleted (C4), Immunologically Quiet (C5), and TGF-β Dominant (C6) [7]. They were characterized by differences in macrophage or lymphocyte signatures, Th1:Th2 cell ratio, extent of intra-tumoral heterogeneity, aneuploidy, extent of neoantigen load, overall cell proliferation, expression of immunomodulatory genes, and prognosis, representing six different category features of the TME. As shown in Figure 4D, there were more C1 and C3 subtypes in the HR subgroup, while there were more C2 and C4 subtypes in LR subgroup.

### 3.6. Immunotherapy Effects of Different Subgroups

To further explain the relationship between risk score and prognosis, TIDE prediction scores were performed on each sample. The higher TIDE represented the greater potential of immune escape, and the worse the clinical benefits of immunotherapy. As shown in Figure 4E, the HR subgroup had a higher TIDE score (*p* < 0.001), indicating that patients of HR were less likely to profit from ICI. Microsatellite instability (MSI, Figure 4H), exclusion (Figure 4G), and dysfunction (Figure 4F) score were also carried out to further investigate factors affecting prognosis. We found that the value of MSI was distinctly higher in the LR subgroup (*p* < 0.01) while the exclusion (*p* < 0.05) and dysfunction (*p* < 0.001) scores were higher in the LR subgroup, which suggested that patients with higher MSI and lower exclusion and dysfunction scores may have a better efficacy of ICI.

### 3.7. The Accuracy of the Prognostic Model

**Figure 5:**
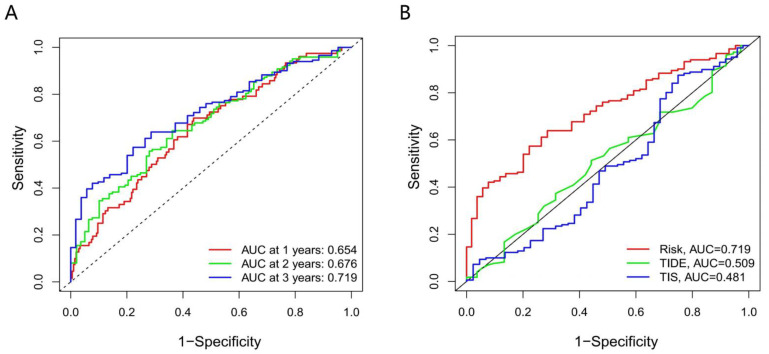
The accuracy of the prognostic model. (**A**) ROC analysis of our model on OS at 1-, 2-, and 3-year follow-up. (**B**) ROC analysis of our model, TIS, and TIDE on OS at 3-year follow-up.

From the ROC curve, we can confidently draw a conclusion that our prognostic model had good performance in survival prediction at 1, 2, and 3 years, with an accuracy of 0.654, 0.676, and 0.719. Furthermore, in terms of AUC, our model (0.719) also preceded the traditional TIDE (0.509) and TIS (0.481) model (Figure 5) at 3 year follow-up.

**Figure 6:**
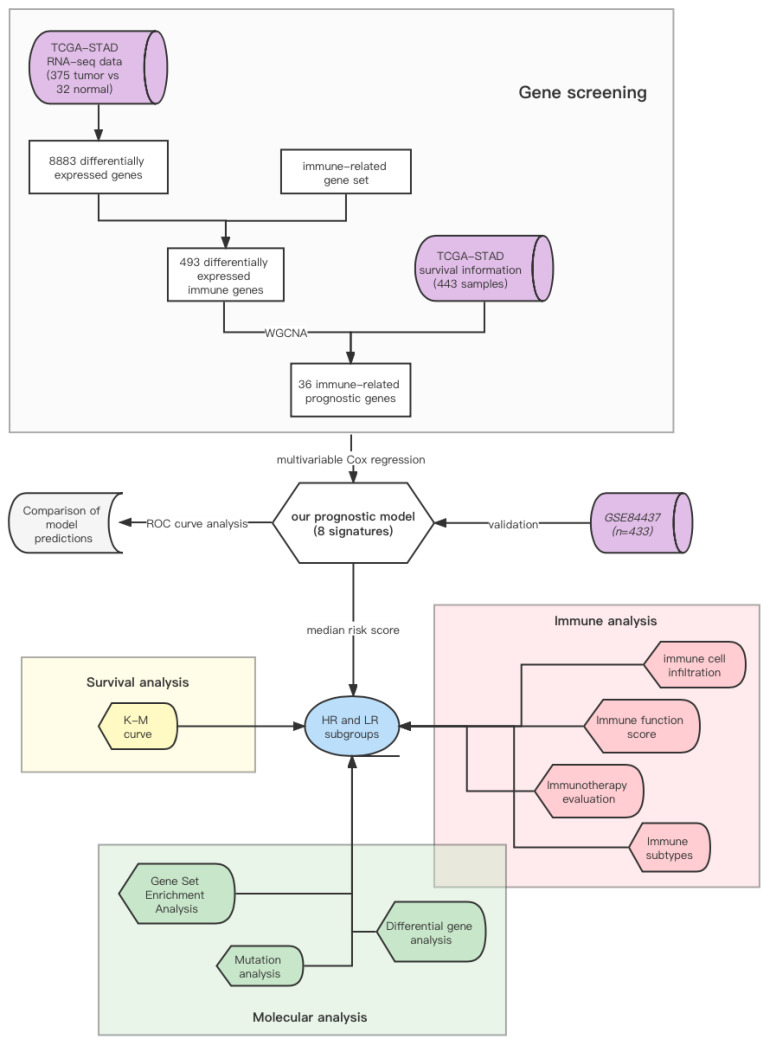
Schematic diagram of construction of Cox regression model based on 8 immune-related genes.

These outcomes showed that our model was a promising prognostic biomarker for patients who are urging for perioperative chemotherapy (Figure 6).

## 4. Discussion

With an increased understanding of the tumor-immune microenvironment, ICI therapy has gradually become a novel treatment for cancers [8,9]. Some PD-1/PD-L1 pathway inhibitors have been proven to be effective for patients with advanced GC and were approved as a third-line therapy in GC [2,10]. However, their use as first-line options is still under evaluation. The objective response rate (ORP) of patients with GC is still low [11]. To circumvent this low ORP, identifying patients who can benefit most from ICI is crucial. Currently, it is acknowledged that different subclasses of the immune environment influenced tumor response to ICI [12]. This highlights the urgency to identify prognostic and immune-therapeutically relevant gene signatures for ICI in GC.

### 4.1. CTLA4 Plays a Vital Role in Response to ICI

Due to the complexity and diversity of the TME, WGCNA is used to identify candidate immune-related biomarkers. With the analysis of the TCGA database, we identified 36 immune-related genes affecting patient prognosis via WGCNA and constructed the model according to eight genes. Among these eight genes, CTLA4, a major negative regulator of T cell responses, has been an apparently highly effective target in the treatment of a variety of highly malignant forms of cancers. Lpilimumab, a monoclonal antibody, activates the immune system by targeting CTLA4 and has been approved by the FDA for the treatment of advanced gastric cancer [13]. It is noted that Lpilimumab does not provide obvious benefit in GC patients but can be used in combination with other ICI or chemotherapy [14]. The combination of anti-PD-1 antibody and Lpilimumab increased response rates and progression-free survival in patients with melanoma, especially in PD-L1-negative patients [15]. These suggested that CTLA4 plays a vital role in response to ICI treatment. Our model showed that CTLA4 is a biomarker positively associated with the LR group, suggesting that patients with a higher level of CTLA4 may respond well to ICI treatment, consistent with the important role of CTLA4 in immune therapy.

### 4.2. Relationship between Immune-Related Genes and Cancers

Bioinformatics analysis showed that RNASE2, an RNA-binding protein, was identified as a novel immune prognostic marker in multiple kinds of cancers [16]. CGB, also known as CGB5, plays an important role in cancer growth, invasion, and metastasis [17]. Especially in tumor-resistant immunity, CGB contributes to desensitization of the immunological system toward cancer cells [18]. RNASE2 and CGB have been reported to be significantly associated with the overall survival of GC patients [19], which is consistent with the basis of our model. The role of INHBE in GC has not been reported. INHBE is upregulated in pancreatic cancer, and it can predict the prognosis of patients with papillary renal cell carcinoma [20]. INHBE may act during drug-induced endoplasmic reticulum stress, but the associated mechanism remains elusive [21]. DUSP1, a “critical node” of the MAPK pathway, acts as a negative regulator of innate immunity and plays a complex role in adaptive immunity [22]. As for adaptive immunity, it negatively regulates JNK and ERK1/2 signaling in T-cells and inhibits induction of regulatory T cells by downregulating TGF-β2 production from dendritic cells [23]. In addition, DUSP1 seemed to impede invariant natural killer T cell activation through MAPK signaling [24]. Despite the prominent mechanisms of the MAPK pathway in cancer, the role of DUSP1 in cancer still remains controversial. However, it is reported that a DUSP1 blockade might provide a novel chemotherapeutic strategy to sensitize cancer cell death [22]. Taken together, given the robust role of DUSP1 in the immune system and chemo-resistance, it is reasonable to serve it as a prognostic marker of immunotherapy. Recently, Ma and colleagues discovered that higher expression of CD36 is associated with shorter survival since CD36-mediated ferroptosis of tumor-infiltrating CD8^+^ T cells restricted antitumor immunity [25]. Shihao Xu also found that CD36 promoted lipid peroxidation and dysfunction in CD8^+^ T cells in tumors [26]. These studies suggested that blocking CD36 may serve as a new immune target, the same as CTLA4.

### 4.3. Somatic Mutation, TMB and TME Corelated with Response to ICI Treatment

Increasing evidence suggests that somatic mutations can produce neoantigens that are able to elicit potent T cell responses driven by current immunotherapies such as ICI [23]. Consistent with these findings, we analyzed gene mutations of different risk groups and found that the mutation rate in the LR subgroup was significantly higher than that in the HR subgroup. This suggests that patients who responded to ICI well had a higher mutation rate. In addition, we found that missense mutation was the most common mutation type, followed by nonsense and frame shift deletions. The largest difference of mutations between groups was TTN mutations, which were more common in the LR group than in the HR subgroup (54% vs. 40%). This was consistent with the studies that patients with mutated TTN had longer progression-free survival than those with wild-type status [27]. Since different types of TP53 mutations were associated with enhanced/reduced immune cell infiltration and PD-L1 expression, which influence the response to ICI [28], the types of TP53 mutation were different in different types of cancer. Thus, the role of TP53 mutations in antitumor immunity is still controversial. For GC, other bioinformatics analyses showed that TP53 mutations inhibited immune response [29], which was inconsistent with our results. However, our model showed that not only the TP53 mutations but also PD-L1 expression was higher than that in the HR subgroup, suggesting that TP53 mutations were positively associated with PD-L1 expressions and response to ICI.

TMB is defined as the total number of somatic genes that code errors, substitutions, base insertions, or deletion errors detected per million bases [30]. Recent studies indicated that TMB, as endogenous antigens, could activate CD8^+^ cytotoxic T cells and trigger T-cell -mediated antitumor activity [31]. Therefore, increased TMB produced more new antigens and enhanced response to immunotherapy, which might predict the response to immunotherapy [32]. Here, we found that risk score had a significant correlation with TMB, which suggested that TMB helped explain the feasibility of our model with immunotherapy prognosis, while there are more other possible mechanisms involved in it.

TME is a hive of immune activity where an array of factors including the intrinsic properties of a tumor and extrinsic factors act to either promote or inhibit anticancer immunity [33]. Among these extrinsic factors, infiltrating immune cells play a crucial role in immunotherapy. In our analysis, the composition of infiltrating immune cells has a significant difference between HR and LR subgroups. Cytotoxic CD8^+^ T cells, activated CD4^+^ T cells and M1 macrophages are enriched in the LR subgroup while M0 and M2 macrophages are more in the HR subgroup. The most frequent subset of CD8^+^ T cells is classical IFN^+^ T cell, which correlates with a more favorable prognosis [34], likely due to cytotoxic potential in conjunction with IFN-gama [35]. A recent clinical study showed that M1 macrophages enhanced T cell-dependent immune responses via regulating PD-L1 [36], suggesting that M1 but not M2 macrophages are helpful in the prognosis of immunotherapy. In summary, these explain the reason the higher levels of CD8^+^ T cells and M1 macrophages in the LR group.

### 4.4. Relationship between Immune Subtypes of Solid Tumors and Our Model

A variety of factors contribute to the response to ICI. According to patient responses to anti-PD-1/PD-L1 therapy, there are three basic immune profiles including immune-inflamed, immune-excluded, and immune-desert phenotype [33]. Patients with immune-inflamed phenotype are characterized by the presence in the parenchyma of CD4^+^ and CD8^+^ T cells, monocytic cells, accompanied by PD-L1, which showed a positive response to ICI. They are completely consistent with the characteristics of the LR subgroup in our model. These immune-infiltrating cells are absent in patients with immune-desert phenotype. The major difference of immunity between immune-inflamed and immune-excluded phenotypes is whether immune cells penetrate the parenchyma of tumors. These two subtypes cannot be distinguished from the composition of immune cells. Meanwhile the immune-excluded subtype has unique molecular characters, such as active TGF-β signaling and FAP^+^ CAF-CXCL12 suppressing immunity [37], which can be distinguished by our model. As levels of TGF-β and CXCL12 are more in the LR subgroup than that in the HR subgroup, there were fewer patients with immune-excluded and immune-desert and more patients with immune-inflamed in the LR group.

Recently, six distinct immune subtypes (C1-C4) of solid tumors from TCGA were identified by Thorsson. It is reported that C1 subtype showed intermediate immune infiltration with M2 macrophages and active TGF-β signaling pathway [7], implying suppressive TME. In contrast, the C2 subtype had a strong CD8^+^ signal and the greatest T cell receptor diversity, implying a favorable immune-activated phenotype. In total, 88% of cases in our model belong to C1 and C2 subtypes. There was more C1 in the HR subgroup but more C2 in the LR subgroup. These results indicated that HR patients were immunosuppressive, while LR patients had active immunity.

### 4.5. MSI and TIDE Support Our Model

Further to PD-L1 and TMB that we have discussed above, microsatellite instability (MSI) is the most validated and clinically used FDA-approved biomarker for ICI response [20]. MSI is defined as hypermutations induced by defective DNA mismatch repair in microsatellite regions. The increased score is positively associated with a higher level of CTL, which renders a more effective antitumor immune response. This is consistent with our results that the LR group had a higher MSI score and a higher likelihood of response to immunotherapy. The TIDE score is associated with T-cell dysfunction in CTL infiltrating tumors and T-cell exclusion in CTL-absent tumors, predicting the prognosis of ICI therapy. In agreement with the TIDE prognosis, our study showed that the HR subgroup had less CTL infiltration and higher TIDE, T-cell dysfunction, and T-cell exclusion score.

## 5. Conclusions

Therefore, we have reason to believe that our model provides a valid prognostic immune-related biomarker for ICI therapy, with better survival in the LR subgroup and worse survival in the HR subgroup. Our model might be a potential prognostic indicator of ICI, which needs more studies.

## Figures and Tables

**Table 1:** Eight prognostic genes were screened out via WCGNA and co-expression analysis.

Source_Reference_ID	Gene Name	Coefficient
NM_00293.4	RNASE2	0.276627343597117
NM_033043.1	CGB5	0.255445846089108
NM_031479.3	INHBE	0.501097892816982
NM_003584.1	DUSP1	0.259804690773917
NM_000039.1	APOA1	0.07098152433948
NM_001001548.1	CD36	0.234091941360161
NM_198712.2	PTGER3	−0.348295793637423
NM_005214.3	CTLA4	−0.341191391278679

## Data Availability

The detailed information of GC samples was downloaded from The Cancer Genome Atlas-Stomach Adenocarcinoma (TCGA-STAD), (https://portal.gdc.cancer.gov). The data of nucleotide mutation in these specimens were also downloaded from TCGA-STAD, Workflow Type: VarScan2 Annotation. The other 433 GC samples used to validate our model (GSE84437) were downloaded from another database, Gene Expression Omnibus (GEO: https://www.ncbi.nlm.nih.gov/geo/). The immune-related genes used in our study were downloaded from the Immunology Database and Analysis Portal (ImmPort) (https://www.immport.org/shared/home) and InnateDB (https://www.innatedb.com/) databases. The immune subtypes of the 371 HNSCC samples were provided by USUC Xena (https://xena.ucsc.edu). The specific data of Immunotherapy outcome-related scores (TIDE, MSI, exclusion, and dysfunction situation) were obtained from Tumor Immune Dysfunction and Exclusion (TIDE: http://tide.dfci.harvard.edu/).

## Supplementary Materials

The following supporting information can be downloaded at: https://www.mdpi.com/article/10.3390/genes13050720/s1, Figure S1: Differentially expressed genes in GC, Figure S2: Results of the weighted gene co-expression network analysis (WGCNA), Figure S3: Mutation research of 36 immune-related prognostic genes, Figure S4: Kaplan–Meier curves of 8 modeling genes, Figure S5: Clinical characteristics of different risk subgroups.
