# Supplementary material for: A Risk Model of Eight Immune-Related Genes Predicting Prognostic Response to Immune Therapies for Gastric Cancer
Source: Genes (Basel). 2022 Apr 20;13(5):720. doi: 10.3390/genes13050720 (PMC9141964; doi:10.3390/genes13050720)
Supplement: Supplementary file 1 [file genes-13-00720-s001.zip › genes-1677878-supplementary.pdf]

Supplementary Materials

1. "WGCNA" methods, firstly, free samples were expurgated and the rest samples were clustered based on the clinical data. Next, the similarity matrix is constructed by calculating the Pearson Coefficient between any two genes. The optimal power value (a soft threshold of $\beta=3$) was obtained by fitting index and average connectivity with power scatter plot to construct adjacency matrix. The adjacency matrix was then transformed into a topological matrix with the topological overlap measure (TOM), and genes and modules were clustered by distance (1-TOM). Finally, gene modules were determined by the dynamic cut tree method and those modules with high similarity were merged (height=0.25). Since the genes in the turquoise modules were the most significant correlation to the clinical trait ($p=4e-34$), they were used to construct the co-expression network (weight threshold>0.3). Their gene expression quantity was then extracted from the tumor samples of TCGA (n=375) and combined with the survival data, using the "limma" package of R. Thus, 36 prognostic IRGs ($p<0.05$) were obtained by univariate COX analysis, and their survival curves were made based on the best cut-off value, obtained by the surv_cutpoint function from "survminer" package of R.

A

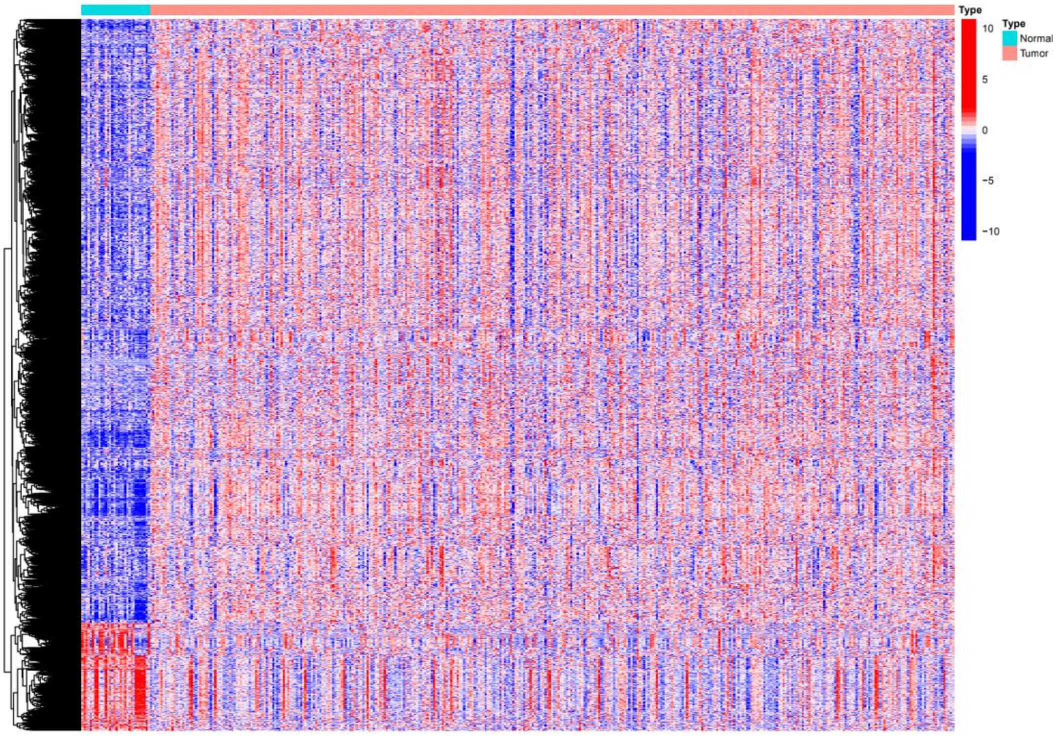

B

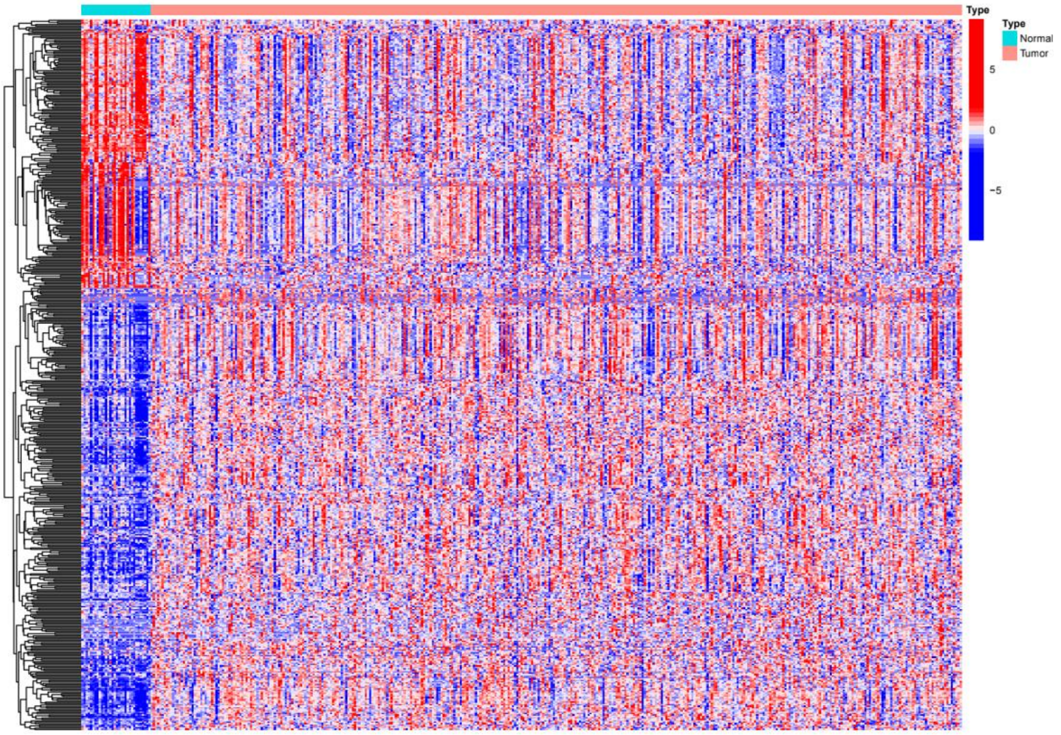

Figure S1. Differentially expressed genes in GC. (A) Heatmap displaying all differentially expressed genes between 375 HNSCC samples (red) and 32 para-cancer samples (blue), 8833 genes in total($p < 0.05$, $|log2FC| > 1$).(B) Heatmap displaying 493 immune-related differentially expressed genes, filtering from the results of Fig.S1A.

A

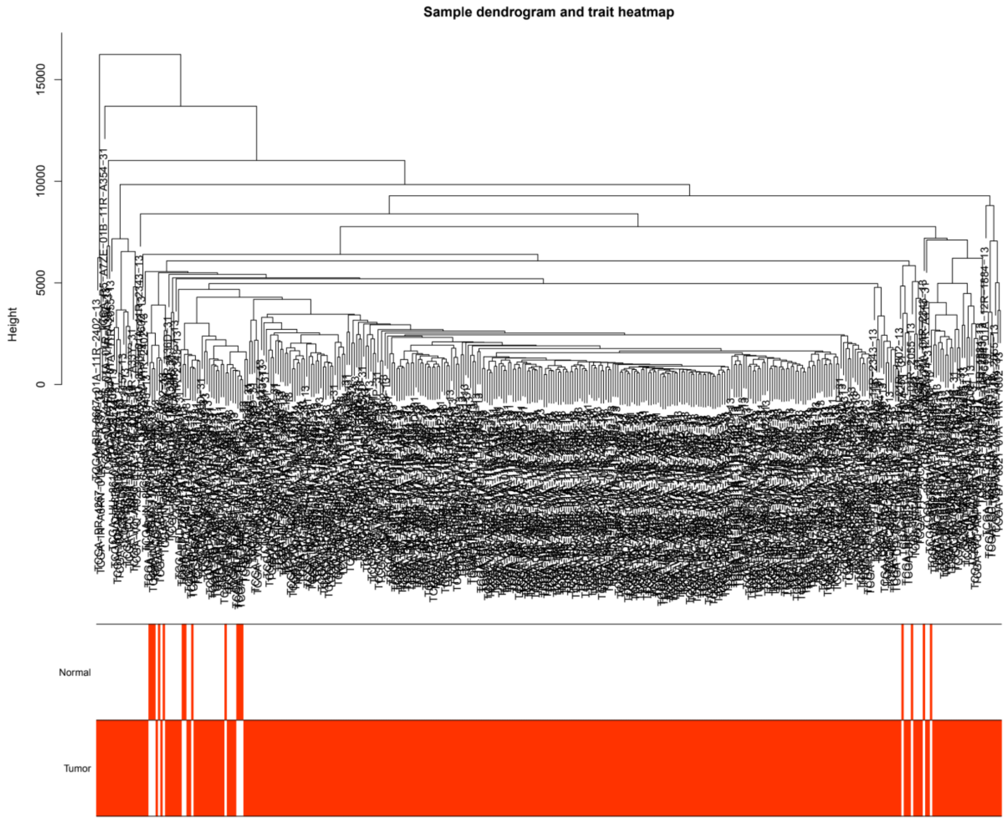
Sample dendrogram and trait heatmap
Height
0
5000
10000
15000
Normal
Tumor

B

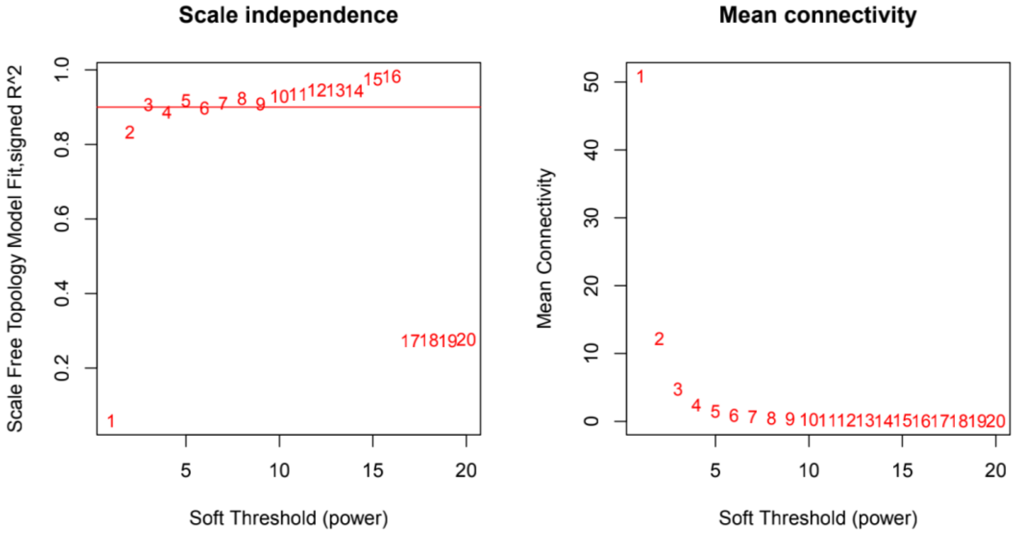
Scale independence
Scale Free Topology Model Fit,signed R^2
Soft Threshold (power)
Mean connectivity
Mean Connectivity
Soft Threshold (power)

C

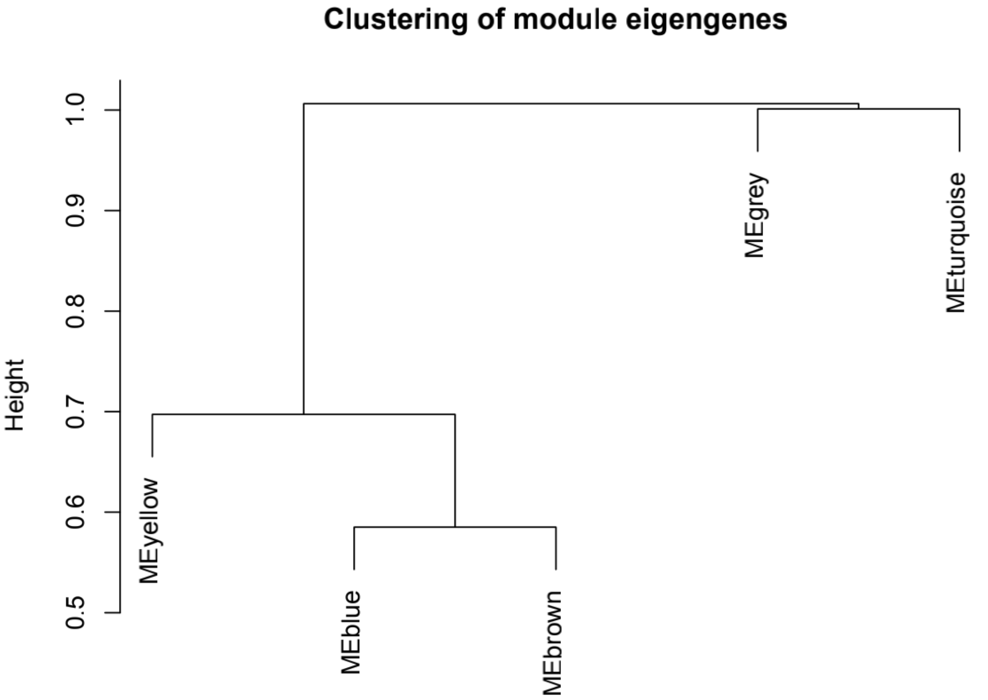

D

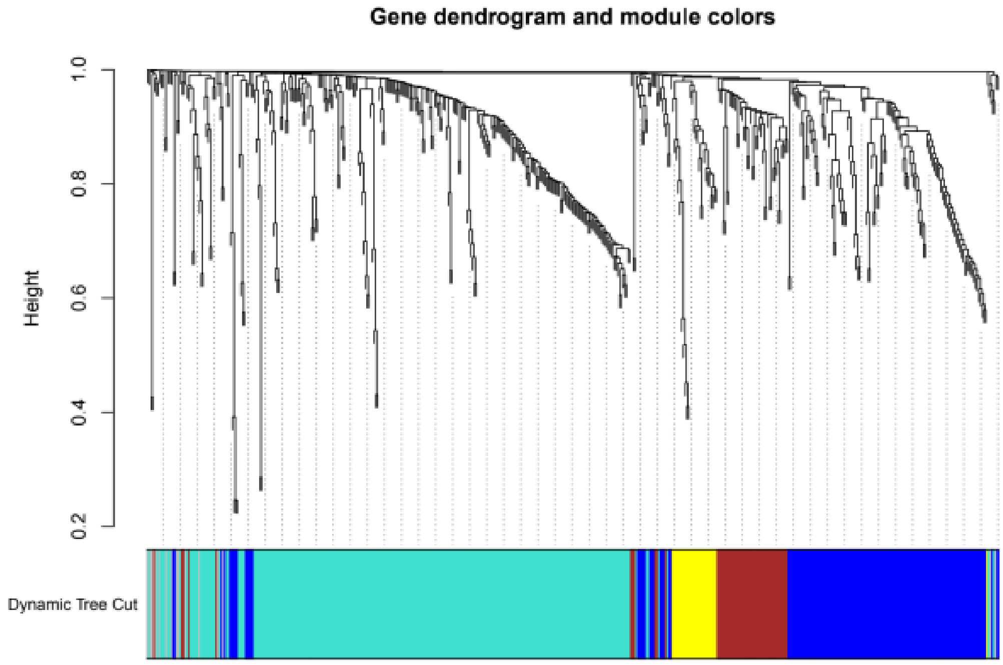

Figure S2. Results of the Weighted gene coexpression network analysis (WGCNA). (A)The sample dendrogram and heatmap shows the clustering and phenotypic information. (B)In the left graph, the horizontal line indicates that the threshold value is 0.90. As seen from the graph, the optimal soft threshold for WGCNA was 3. (C)5 eventual network modules are obtained after merging.(Height = 0.25). (D)The merged graphical result shows the final clustering of samples under different network modules.

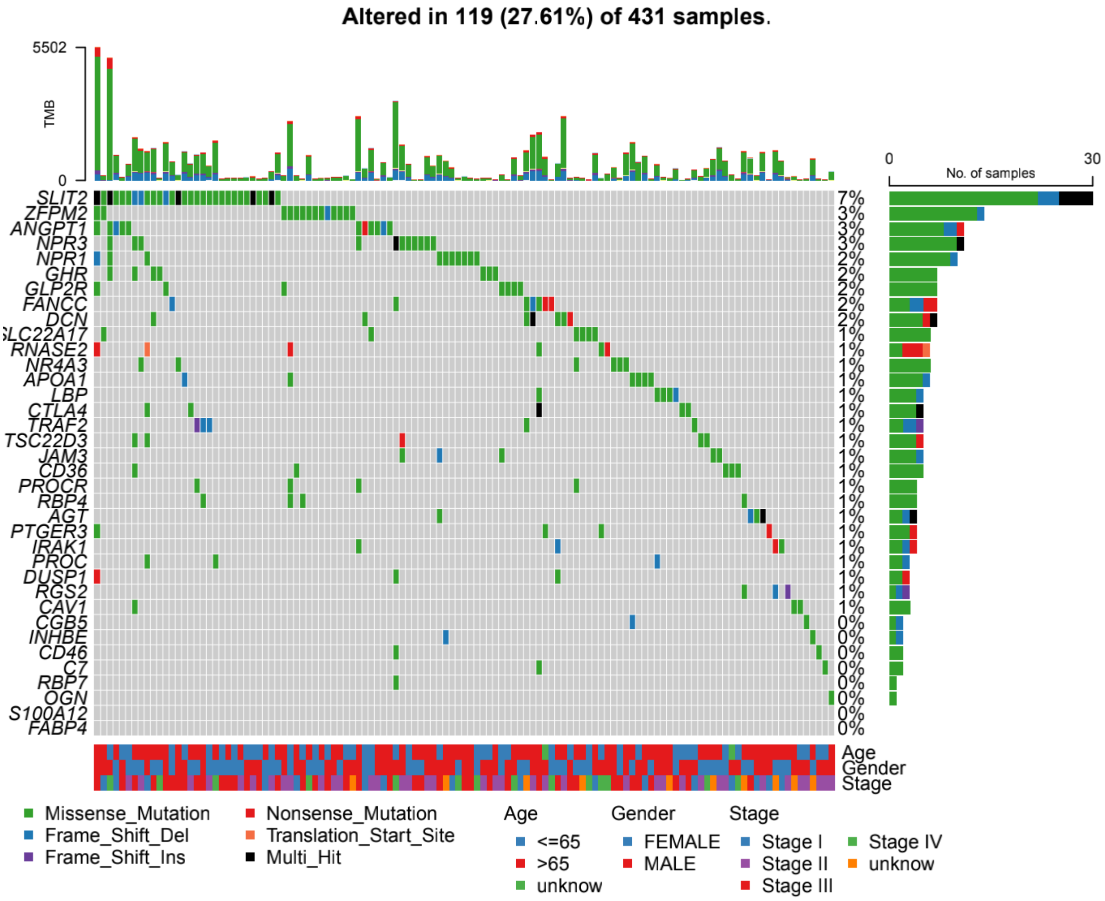

Figure S3. Mutation research of 36 immune-related prognostic genes. The mutation rate of 36 immune-related prognostic gene in TCGA cohort (n = 431).

A

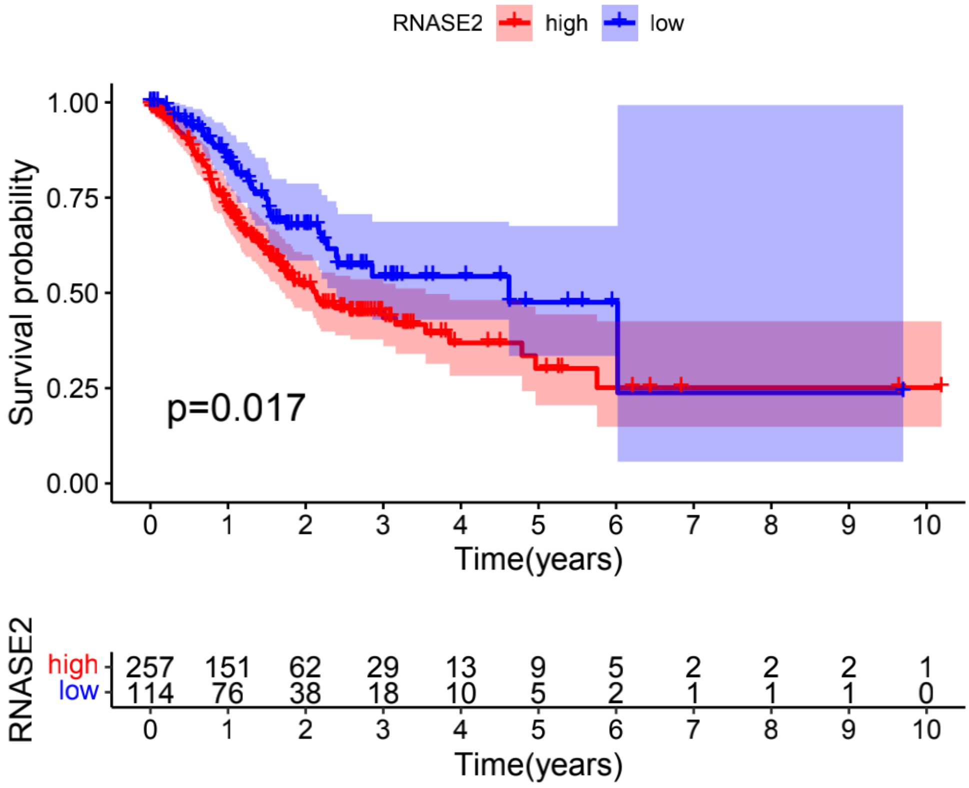

RNASE2
high
low
1.00
0.75
0.50
0.25
0.00
Survival probability
p=0.017
0 1 2 3 4 5 6 7 8 9 10
Time(years)
RNASE2
high 257 151 62 29 13 9 5 2 2 2 1
low 114 76 38 18 10 5 2 1 1 1 0
0 1 2 3 4 5 6 7 8 9 10
Time(years)

B

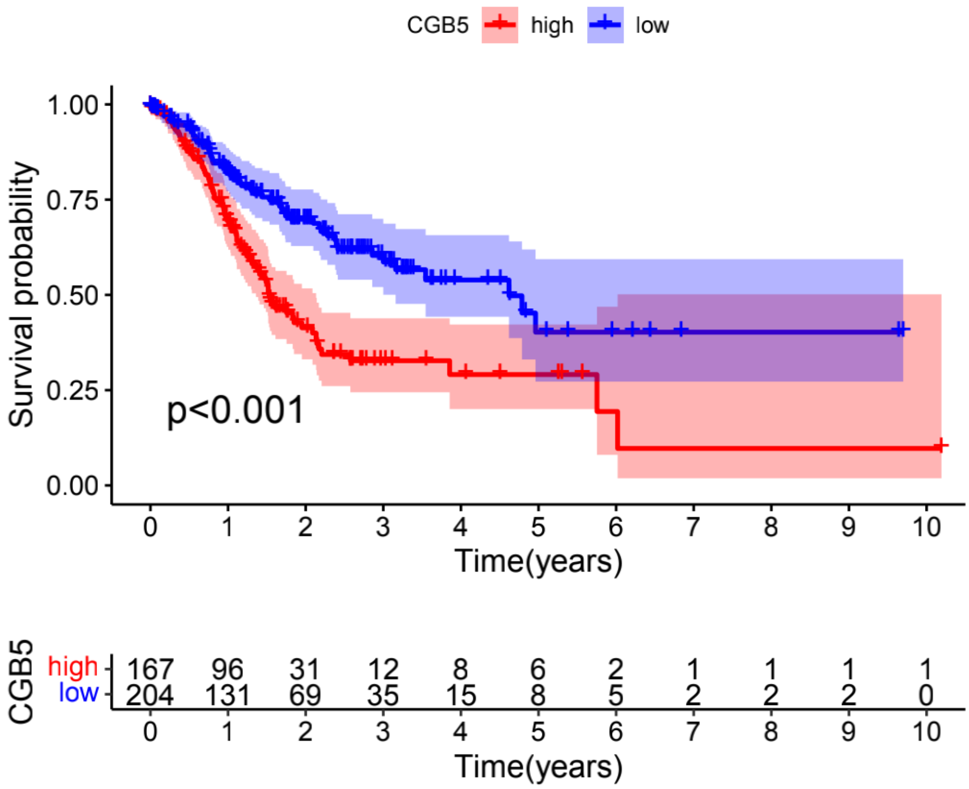

CGB5
high
low
1.00
0.75
0.50
0.25
0.00
Survival probability
p<0.001
0 1 2 3 4 5 6 7 8 9 10
Time(years)
CGB5
high 167 96 31 12 8 6 2 1 1 1 1
low 204 131 69 35 15 8 5 2 2 2 0
0 1 2 3 4 5 6 7 8 9 10
Time(years)

C

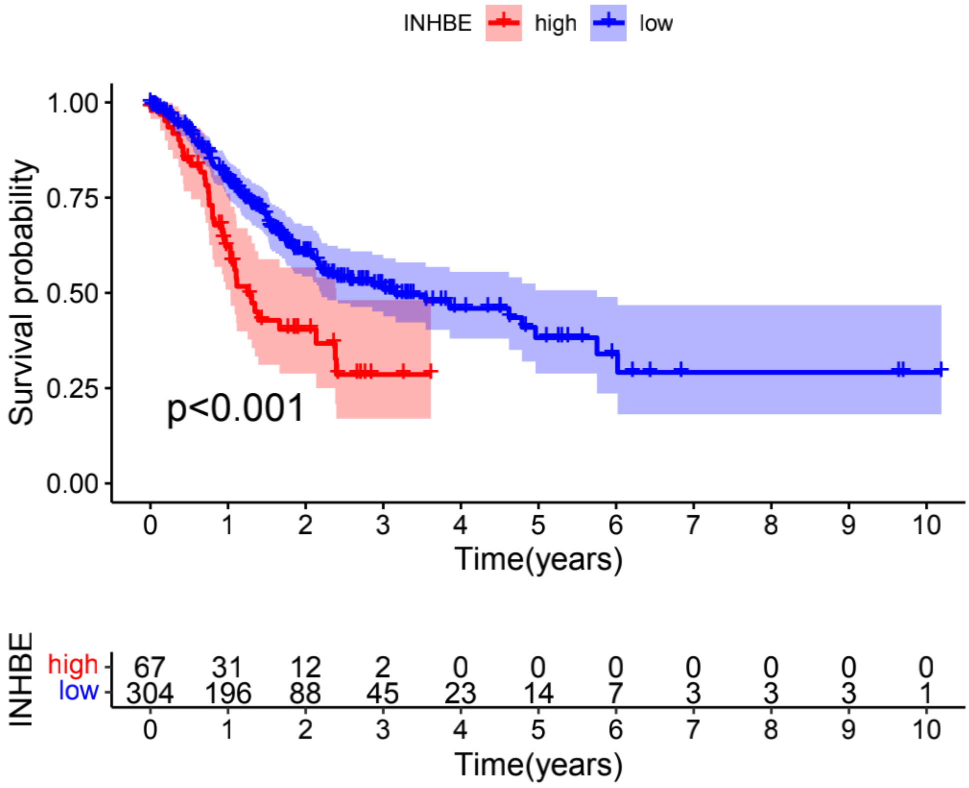
INHBE high low
Survival probability
1.00
0.75
0.50
0.25
0.00
p<0.001
0 1 2 3 4 5 6 7 8 9 10
Time(years)
INHBE
high 67 31 12 2 0 0 0 0 0 0 0
low 304 196 88 45 23 14 7 3 3 3 1
0 1 2 3 4 5 6 7 8 9 10
Time(years)

D

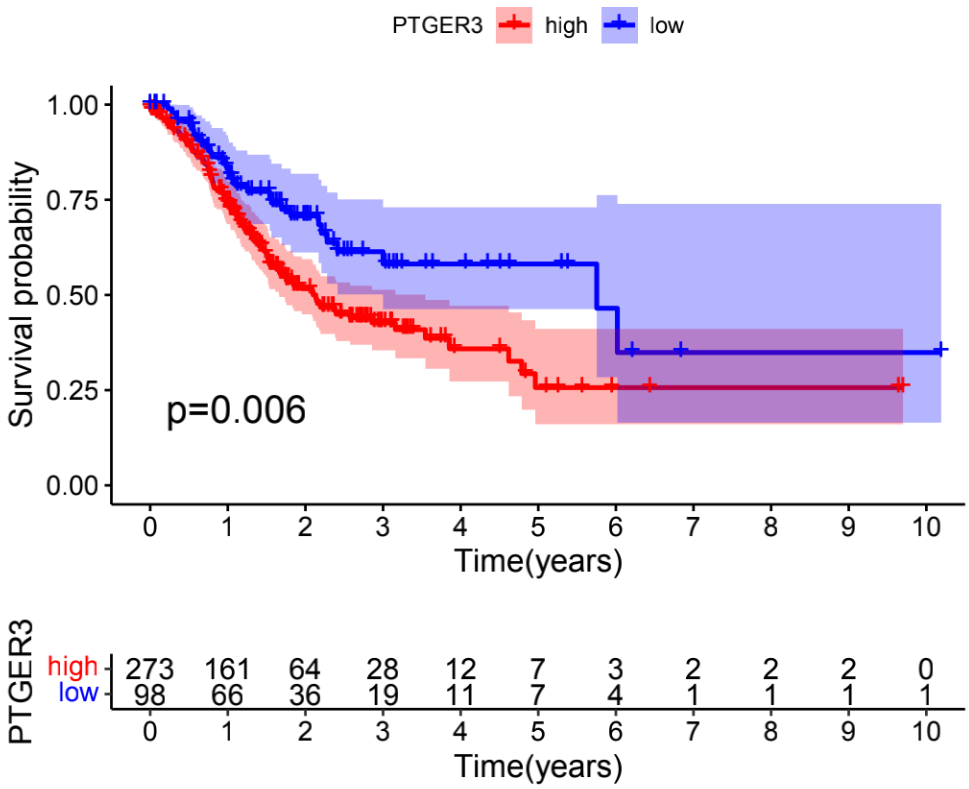
PTGER3 high low
Survival probability
1.00
0.75
0.50
0.25
0.00
p=0.006
0 1 2 3 4 5 6 7 8 9 10
Time(years)
PTGER3
high 273 161 64 28 12 7 3 2 2 2 0
low 98 66 36 19 11 7 4 1 1 1 1
0 1 2 3 4 5 6 7 8 9 10
Time(years)

E

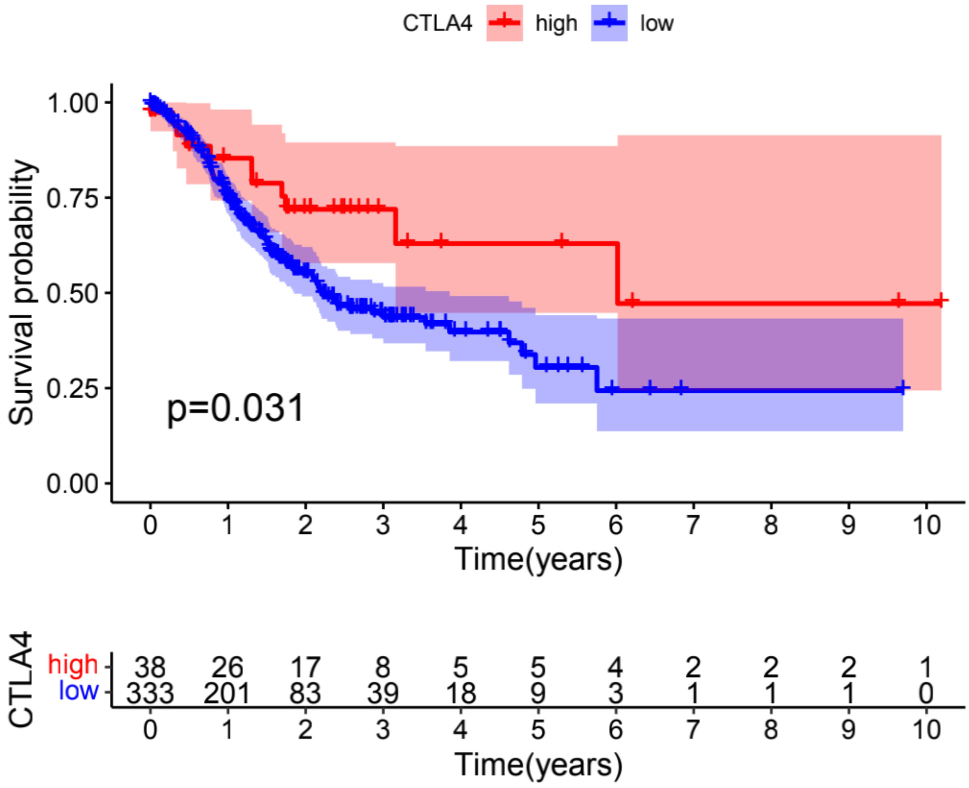

CTLA4
high
low
Survival probability
1.00
0.75
0.50
0.25
0.00
p=0.031
0 1 2 3 4 5 6 7 8 9 10
Time(years)
CTLA4
high 38 26 17 8 5 5 4 2 2 2 1
low 333 201 83 39 18 9 3 1 1 1 0
0 1 2 3 4 5 6 7 8 9 10
Time(years)

F

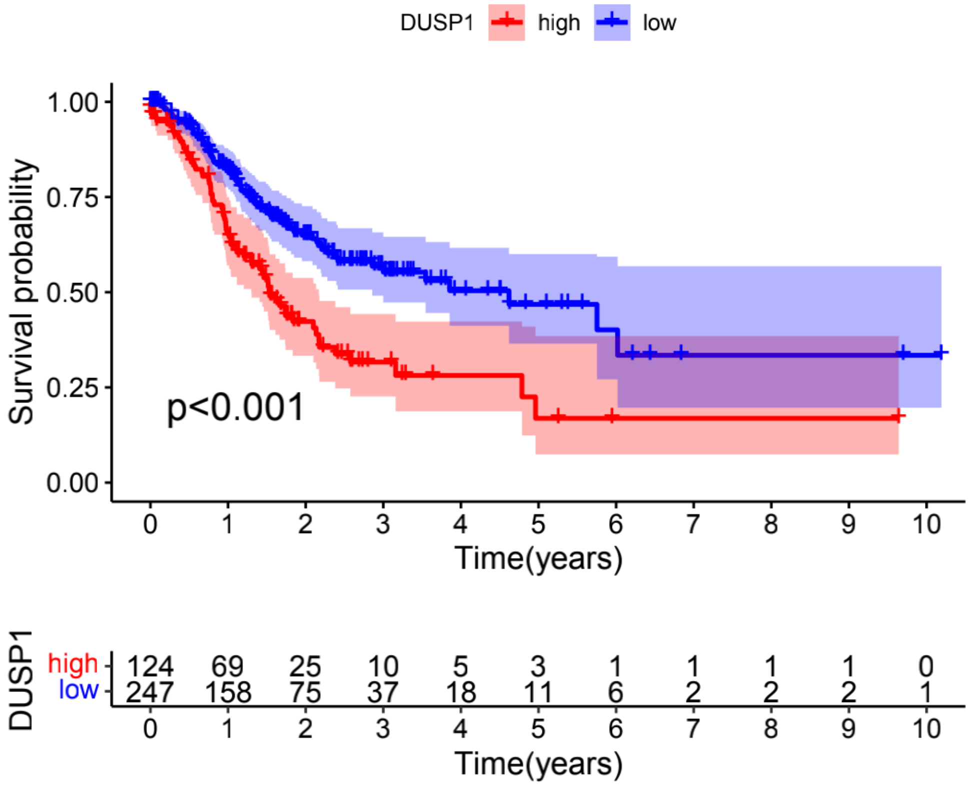

DUSP1
high
low
Survival probability
1.00
0.75
0.50
0.25
0.00
p<0.001
0 1 2 3 4 5 6 7 8 9 10
Time(years)
DUSP1
high 124 69 25 10 5 3 1 1 1 1 0
low 247 158 75 37 18 11 6 2 2 2 1
0 1 2 3 4 5 6 7 8 9 10
Time(years)

G

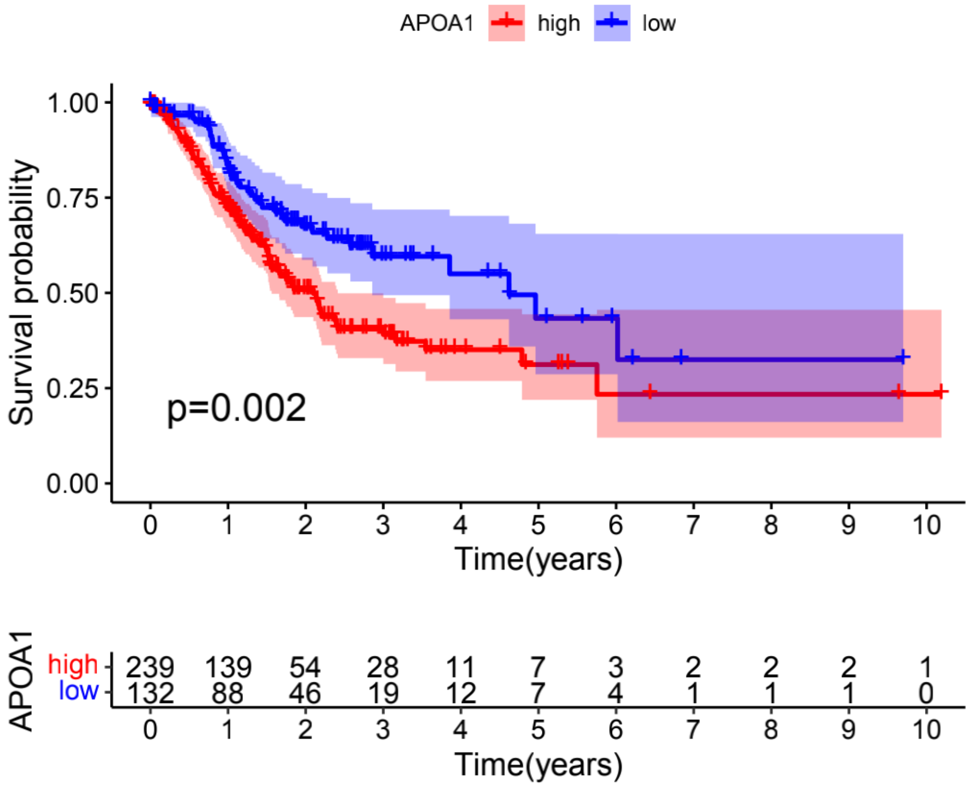

H

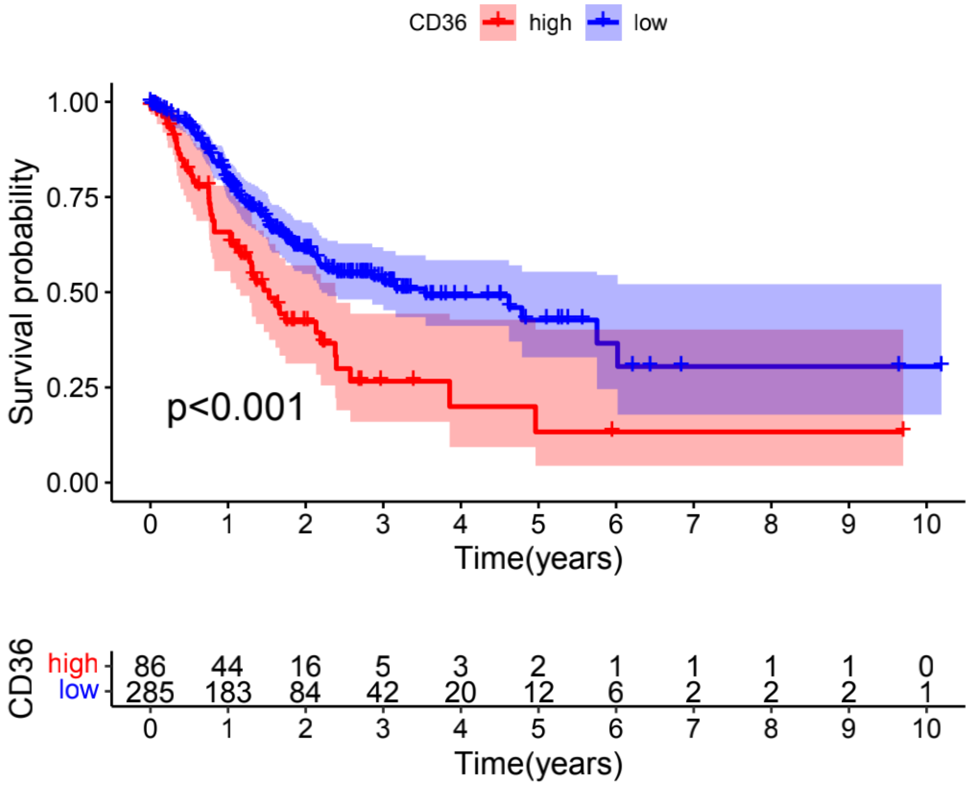

Figure S4. Kaplan-Meier curves of 8 modeling genes. K–M survival analysis of the 8 modeling genes in TCGA cohort. (A) RNASE2 ($p = 0.017$). (B) CGB5 ($p < 0.001$). (C) INHBE ($p < 0.05$). (D) PTGER3 ($p = 0.006$). (E) CTLA4 ($p = 0.031$). (F) DUSP1 ($p < 0.001$). (G) APOA1 ($p = 0.002$). (H) CD36 ($p < 0.001$).

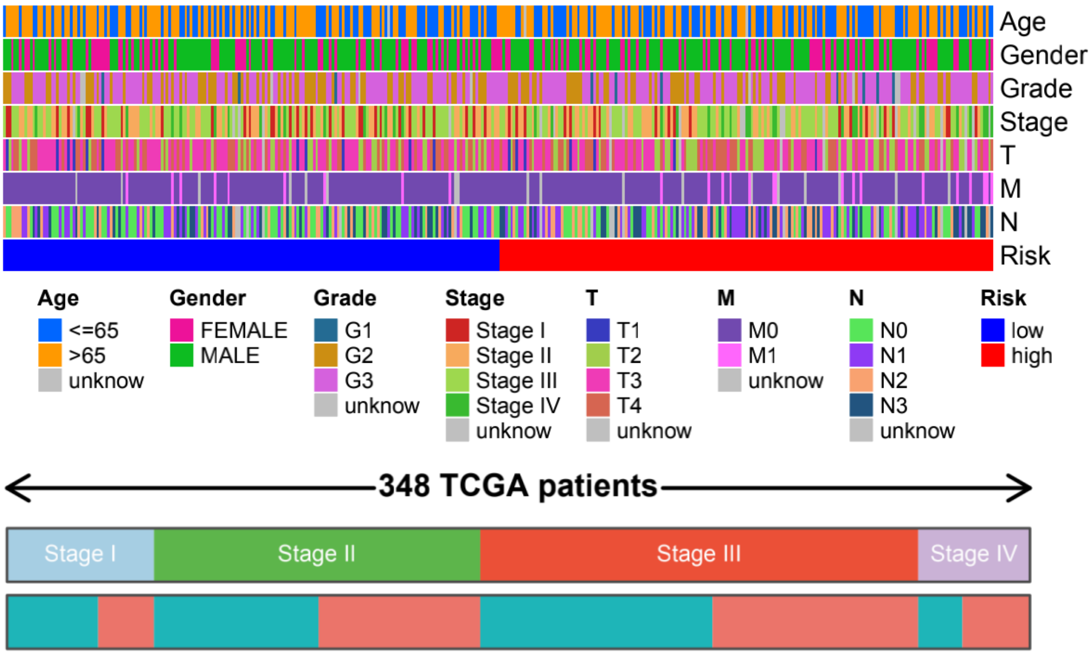

risk score subgroups	Stage group (n=348)				
	Stage I (n=50, 14%)	Stage II (n=111, 32%)	Stage III (n=149, 43%)	Stage IV (n=38, 11%)	*P-value*
low-risk (n=181)	31(17%)	56(31%)	79(44%)	15(8%)	0.207
high-risk (n=167)	19(11%)	55(33%)	70(42%)	23(14%)	

■ low-risk ■ high-risk

Figure S5. Clinical characteristics of different risk subgroups.
